# Contiguous Multilevel Thoracic Ossification of Ligamentum Flavum in a Young Adult Spine

**DOI:** 10.1155/2019/1640485

**Published:** 2019-07-09

**Authors:** Tsuyoshi Yamada, Ichiro Torigoe, Kenichiro Sakai, Atsushi Okawa, Yoshiyasu Arai

**Affiliations:** ^1^Department of Orthopaedic Surgery, Saiseikai Kawaguchi General Hospital, Saitama 332-8558, Japan; ^2^Department of Orthopaedic Surgery, Graduate School, Tokyo Medical and Dental University, 1-5-45 Yushima, Bunkyo-ku, Tokyo 113-8510, Japan

## Abstract

The ossification of the ligamentum flavum (OLF) was characterized by the replacement of the ligamentum flavum by ectopic new bone formation. Although OLF is well known as one of the causes of thoracic myelopathy through the compression of the spinal cord from the posterolateral side, contiguous multilevel OLF is quite a rare cause of myelopathy. Severe ossifications were reported that only present in individuals aged over 25 years. External triggers of OLF might likely be increased as a consequence of biomechanical stress to the effect of posttraumatic ossification. The authors described one rare case of an enormous contiguous multilevel OLF in a 20-year-old female's thoracic spine after mild back trauma, to the extent that it was misdiagnosed as an old epidural hematoma initially. This female with obesity presented with a 6-month history of progressively worsening dorsal cord disorders. Resection or floating of the enormous OLF could be successfully achieved using O-arm-based navigation, and sensory loss, numbness, and gait disturbance were improved after operation.

## 1. Introduction

The ligamentum flavum may undergo various pathologic changes including hypertrophy, calcification, cyst formation, hematoma, and ossification [1, 2]. The ossification of the ligamentum flavum (OLF) was characterized by the replacement of the ligamentum flavum by ectopic new bone formation, and the most frequent OLF lesion is the thoracic spine. Although OLF is well known as one of the causes of thoracic myelopathy through the compression of the spinal cord from the posterolateral side [3], contiguous multilevel OLF is quite a rare cause of myelopathy, reported in a few cases [4–6].

Here, the authors described one case of an enormous contiguous multilevel OLF after mild back trauma, to the extent that it was misdiagnosed as an old epidural hematoma initially. Despite the high rate of perioperative complications in OLF resection, resection or floating of the enormous OLF could be successfully achieved using O-arm-based navigation in the present case.

## 2. Case Presentation

A 20-year-old female with obesity (body mass index: 33.3 kg/m^2^) presented with a 6-month history of progressively worsening paresthesia of the lower limbs and gait disturbance. Moderate muscle weakness, severe spasticity in the lower limbs, a superficial hypoesthesia below the Th6 level, and severe dorsal cord disorders were noted on the first physical examination. There was a history of a contusion in the back following the drop from 2 meters height, treated with conservative therapy 2 years previously. She showed no feature of skeletal dysplasia. Laboratory data revealed no abnormality.

**Figure 1:**
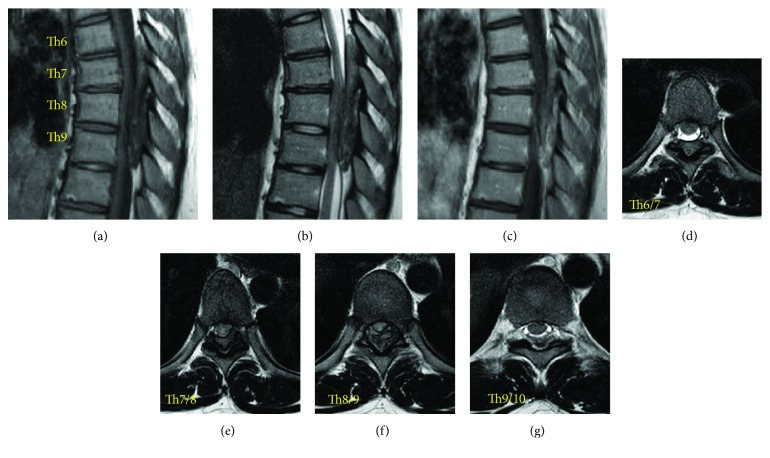
Preoperative thoracic MRI. Heterogeneous epidural posterior mass compressing the thoracic cord between Th6 and Th9 was revealed at the admission. Sagittal view of heterogeneous isointense mass of T1-weighted image (a), heterogeneous isointense mass of T2-weighted image (b), and nonenhancing mass of Gd-enhanced T1-weighted image (c). Axial view of epidural mass of T2-weighted image in Th6/7 (d), Th7/8 (e), Th8/9 (f), and Th9/10 (g) intervertebral levels. This epidural mass was considered as an old epidural hematoma initially.

**Figure 2:**
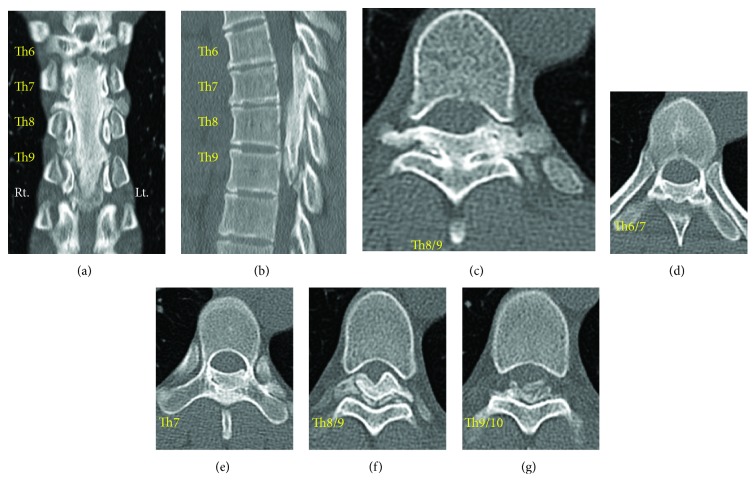
Preoperative thoracic CT. Ossified epidural posterior mass compressing the thoracic cord between Th6 and Th9 was revealed in coronal view (a) and sagittal view (b) of thoracic CT. Ossified lesion reached to extraforamen at Th7/8 intervertebral levels (c). Axial view of epidural ossified mass in Th6/7 (d), Th7 (e), Th8/9 (f), and Th9/10 (g) levels, which occupied about 50-80% of the spinal canal.

Magnetic resonance imaging (MRI) showed a dorsally located epidural lesion (Th6-Th9) which seemed a heterogeneous mass hypointense on both T1- and T2-weighted images (Figure 1). These clinical courses and radiological findings suggested old epidural hematoma. Computerized tomography (CT) scans revealed that this dorsally located epidural lesion consisted of ossified components, which occupied about 50-80% of the spinal canal, and partially developed even to extraforaminal regions (bilateral Th7/8 foramen) or to the extent which integrated the lamina (Th7-Th8 laminas), leading to make the spinal cord compressed severely. These ossification lesions seemed to have the bone marrow inside (Figure 2).

**Figure 3:**
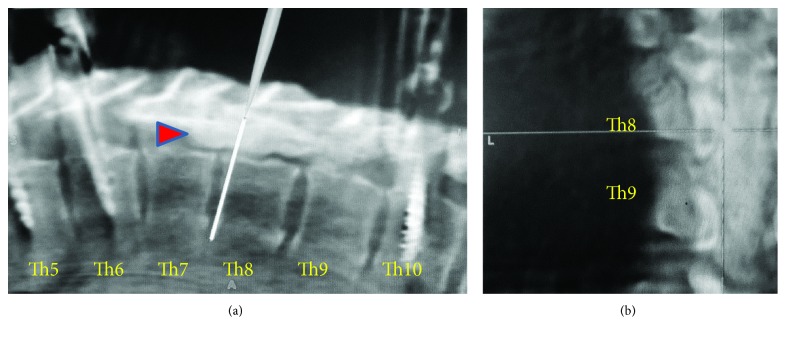
Surgical excision of ossified lesion under O-arm-based navigation system. After performing Th6-Th10 laminectomy, the posterior epidural space was filled with an ossified mass. To float the adhered ossified mass, pediculectomy of Th7 and Th8 and posterior spinal fusion from Th5 to Th11 were performed. Surgeons can resect or float an enormous contiguous ossified mass safely (arrowhead), confirming the thickness of ossification and the adhesion to circumferential tissues under O-arm-based 3D navigation (sagittal view (a), coronal view (b)).

**Figure 4:**
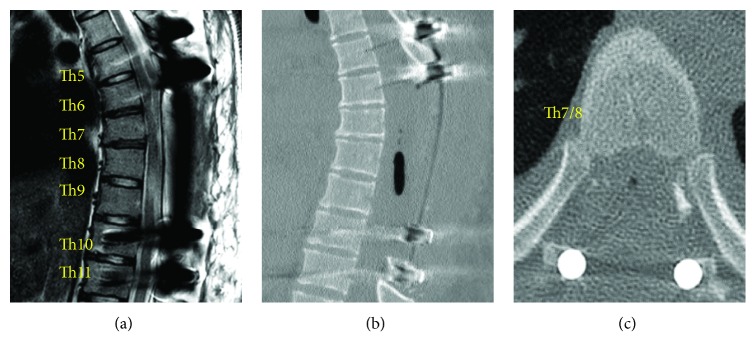
Thoracic spine radiological findings post Th5-Th11 posterior spinal fusion. Postoperative MRI showed the dura matter swollen (sagittal view (a)), while CT demonstrated the total resection of these extremely ossified components (sagittal view (b), axial view in Th7/8 level (c)).

Thoracic pedicle screw insertions in Th5, 6, 10, and 11 levels preceding thoracic cord decompression and the surgical excision of ossified lesion or floating of ossified lesion with the slight adhesion to dura matter were performed under O-arm-based navigation system (O-arm Surgical Imaging System and StealthStation; Medtronic, Inc., Minneapolis, MN, USA). When Th6-Th10 laminectomy was performed, the posterior epidural space was filled with an ossified mass. Between Th6-Th9 laminas and the ossified mass, there was a part of denatured flavum with abundant blood vessels. In particular, these ossified lesions developed in Th7-Th8 levels, where this mass adhered to bilateral pedicles of Th7 and Th8 (Figure 3). To float these lesions, pediculectomy of Th7 and Th8 was performed. We found bilateral OLF in Th9/10 level, separated from Th6-9 epidural ossified mass. This separated beak lesion was considered as the dynamic factor to mainly cause thoracic myelopathy through the compression of the spinal cord. The resection or floating of these extremely ossified components made compressive dura matter swollen (Figure 4). After the resection of ossified lesions, an autologous local bone graft harvested during decompressive laminectomy was placed between decorticated transverse processes or remained laminas as posterolateral spinal fusion from Th5 to Th11. The operation took 303 minutes. In spite of denatured enormous lesions, the intraoperative blood loss was no more than 420 mL, by repeating vigorous hemostasis by electrocoagulation, bone wax, and FLOSEAL hemostatic matrix (Baxter Healthcare Corp, Fremont, CA, USA).

**Figure 5:**
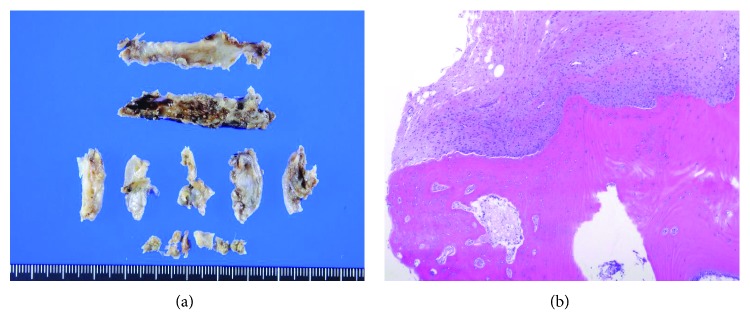
Histopathological analysis of the resected tumors. Macroscopic findings of the resected epidural mass (a). Ossification of the ligament tissue was observed in the resected tumor. This mass was diagnosed as a contiguous multilevel OLF according to microscopic findings (hematoxylin and eosin stained) (b).

Ossification of the ligament tissue was observed in the resected tumor. Contiguous multilevel OLF was diagnosed and confirmed by pathology (Figure 5). After operation, sensory loss, numbness, and gait disturbance were improved. Her Japanese Orthopaedic Association (JOA) score for thoracic myelopathy recovered from preoperative 5.0 points to 8.0 points out of 11 points. Following examinations indicated the absence of recurrence or neurological deterioration.

## 3. Discussion

The onset of OLF-related symptoms with consecutive surgical treatment ranges between 55 and 65 years, as the prevalence increases with age. The formation of OLF in the population is strongly considered due to degenerative wear-and-tear. There are reports on OLF manifestations in relation to diabetes mellitus, activity patterns (heavy manual labour in males), body weight (high-BMI values in females), fluorosis, increased bone density, hyperparathyroidism, diffuse idiopathic skeletal hyperostosis (DISH), ankylosing spondylitis, acromegaly, and achondroplasia [7]. External triggers of OLF might likely to be increased as a consequence of biomechanical stress to the effect of posttraumatic ossification [8].

OLF can significantly contribute to a spatial reduction of the thoracic spinal canal, resulting in slowly progressive or acute paraplegia after trauma to the back. Given a 2-year-ago history of trauma and her young age, the formation of OLF in the present case could be attributed to the effect of posttraumatic ossification, in addition to the influence of intrinsic high-BMI values. Because of the difficulty in clinically monitoring contusion in the back without fracture or neurological deterioration when we take the attributed risks of imaging radiation exposure and medical expenses into consideration, the exact incidence of epidural hematoma and much more epidural hematoma ossification is unknown. Although the posttraumatic or postoperative ossified epidural hematomas in cranial, especially in children, were discussed in a few reports [9–11], the thoracic spine lesion has not been reported. Differential diagnosis must include some epidural tumors and spinal epidural hematomas. All the cases of spinal epidural hematomas which needed emergent surgery in our institute (8 cases, from 2010 to 2016) showed hypointense mass on T1-weighted imaging while the intensity on T2-weighted images seemed variable. The epidural mass in this case was misdiagnosed as an old epidural hematoma initially, based on MRI findings. The diagnosis of this ossified mass had not been unconfirmed until we saw the result of pathology.

According to the previous classification on axial CT scan and T2-weighted sagittal MRI image [12], we could regard the present thoracic OLF in a young adulthood as bridged and round types. These bridged and round ossifications reached from Th6 to Th9 levels, shaping an enormous contiguous multilevel OLF with thickness over 4 mm. Grade 3 (≧4 mm) ossifications were reported that only present in individuals aged over 25 years, and a later progression of the condition that would almost exclusively have occurred in old age (≧46 years) [7]. To our knowledge, we present the first case of such an enormous contiguous multilevel OLF in a young adult thoracic spine, to the extent which myelopathy advanced to need posterior decompression and fusion largely.

Thoracic myelopathy secondary to OLF can be treated with laminectomy. Further, laminectomy with partially internal fixation is safe and effective in treatment of patients with symptomatic multilevel OLF [5]. The overall perioperative complication rate reached to 35%, with dural tears and cerebrospinal fluid leaks most frequently [13]. In the present case, we could successfully avoid such a complication because we could resect or float an enormous contiguous OLF safely, confirming the thickness of ossification and the adhesion to circumferential tissues under O-arm-based navigation. The use of intraoperative navigation in spine surgery, particularly the O-arm, has increased rapidly in the past several years, and many authors has addressed the utility of the O-arm [14]. In the case of a contiguous multilevel OLF, O-arm-based 3D navigation is one of the safe and effective measures during surgery.

The authors described quite a rare case of an enormous contiguous multilevel OLF in young female thoracic spine after mild back trauma. Asymptomatic chronic epidural hematomas even in young adult spine might undergo large ossification, and enormous OLF could prevent natural absorption, leading to cause myelopathy. If there is a chronic spinal epidural mass with ossification, we recommend surgery before irreversible change has been completed. The use of O-arm-based intraoperative 3D scans for navigation can make drilling and resection of an enormous contiguous OLF reliable.
